# Stable Coloured Micrometric Films from Highly Concentrated Nano-Silver Sols: The Role of the Stabilizing Agents

**DOI:** 10.3390/nano11040980

**Published:** 2021-04-10

**Authors:** Eleonora Pargoletti, Marco Aldo Ortenzi, Giuseppe Cappelletti

**Affiliations:** 1 Dipartimento di Chimica, Università degli Studi di Milano, Via Golgi 19, 20133 Milan, Italy; eleonora.pargoletti@unimi.it (E.P.); marco.ortenzi@unimi.it (M.A.O.); 2 Consorzio Interuniversitario per la Scienza e Tecnologia dei Materiali (INSTM), Via Giusti 9, 50121 Firenze, Italy; 3 CRC Materiali Polimerici “LaMPo”, Dipartimento di Chimica, Università degli Studi di Milano, Via Golgi 19, 20133 Milano, Italy

**Keywords:** silver nanoparticles, concentrated sols, capping agents, colored micrometric films, UV stability

## Abstract

The synthesis of highly concentrated aqueous silver nanoparticles (NPs), exploiting different types of polymeric stabilizing agents, has been extensively investigated, especially for the stabilization of spherical yellow nanoparticles. In this context, here, a successful and easy wet chemical method was adopted to synthesize concentrated primary colored (yellow, red, blue and green) sols. The influence of polyvinylpyrrolidone (PVP) and polyvinyl alcohol (PVA) in affecting the final stability was finely investigating via UV/Vis spectroscopy, dynamic light scattering, TEM and colorimetric analysis. The next step consisted on the deposition of obtained sols onto a crown-treated polyethylene terephthalate (PET) support to obtain transparent colored micrometric homogeneous films. The fabricated PVP-based Ag films were revealed to be outstandingly UV-stable, contrarily to PVA-based films, probably due to the degradation of the polymer itself. Indeed, after UV aging tests, the PVA macromolecules could be broken and chemically modified (demonstrated by FT-IR analyses). This resulted in there being insufficient macromolecules to efficiently cover the surface of the nanoparticles, meaning that the nanoparticles tended to aggregate with each other, destabilizing the system itself. Hence, the obtained colored films described herein could represent a promising tool for different applications, from color shifting to optoelectronic devices.

## 1. Introduction

In recent years, the synthesis of nanomaterials with well-defined shapes and sizes has stimulated great scientific and technological interest because of their fascinating and unique optoelectronic [1], magnetic [2] and catalytic [3] properties. Specifically, silver nanoparticles (NPs) have received particular interest due to their promising performances in several applications, such as tunable localized surface plasmon resonance (LSPR), surface enhanced Raman scattering, biosensors, printed electronics, and antimicrobial technology [1,4,5,6,7]. Concerning the LSPR phenomenon, colloidal systems comprising of noble metal nanoparticles (such as Au or Ag), usually generate a wide range of colors thanks to the effects of light absorption and dispersion in the visible region (380 to 750 nm). Notably, such wavelength range is strictly related not only to the refractive index of the medium, but also to the morphology and size of the NPs (plasmonic effect) [8]. Over recent decades, several research groups have described synthetic routes for the creation of aqueous colloidal Ag nanoparticles by reacting silver salt precursors (such as silver nitrate) with a reducing agent (as sodium borohydride or ascorbic acid) in the presence of a stabilizer [9,10,11]. Remarkably, as regards the last compound, several polymers have been investigated so far. Anderson et al. [10,12] reported the synthesis of highly concentrated (>1 M) yellow spherical Ag NPs by exploiting a methoxypoly(ethylene glycol) acrylate and maleic anhydride (PEG-MA) blend. Other studies have revealed that the binding ability of water-soluble polymers, such as polyvinylpyrrolidone (PVP) and polyvinyl alcohol (PVA), to the silver surface represents the tool to control the nucleation, growth and shape of the final NPs [13,14,15]. However, the main problem often encountered during the synthetic process, is nanoparticle aggregation upon increasing the concentrations of the Ag precursors [1,4]. Shahzad and co-workers [4] recently developed an alternative aqueous-phase method for the preparation of highly concentrated (above 20 g L^−1^) yellow Ag nanoparticles with long-term stability and a size of about 8 nm, exploiting branched polyethyleneimine (BPEI) as a multifunctional polymer. However, the engineering of a simple synthesis method for highly concentrated metal nanoparticles with different morphologies still has room for improvement.

Hence, we propose a very easy method to synthesize Ag NPs with concentrations (up to 330 μM) slightly higher than those previously reported (about 200 μM for green, blue and red colors [16,17]), resulting in very stable sols, thanks to the controlled shape and size of the NPs via the use of PVP or PVA capping agents. In particular, yellow concentrated sols (up to ca. 2 M [12]) have already been largely described; whereas an optimized synthesis method for the other concentrated (200 μM) Ag colors has not been reported so far, due to the great instability of these sols. Furthermore, herein, the next step forward is the successful deposition of these concentrated sols onto a crown-treated polyethylene terephthalate (PET) support, in order to develop transparent colored micrometric films, preserving the initial color of Ag sols and limiting particle sintering during the solvent evaporation. As such, the prepared films can be employed in several application fields, e.g., from color shifting [4,7] to optoelectronic devices [1] and antibacterial food packaging [18].

## 2. Materials and Methods

All the chemicals were of reagent-grade purity and were used without further purification. Doubly distilled water passed through a MilliQ apparatus (Merck Millipore, Burlington, MA, USA) was utilized.

### 2.1. Synthesis of Diluted and Concentrated Ag Nanoparticles (NPs)

For both diluted and concentrated silver sols, the adopted synthetic route comprised the preparation of yellow silver seeds to be further used for red, blue and green colors. Concerning the diluted aqueous systems, they were synthesized by reducing silver nitrate 10 mM (AgNO_3_, purchased from Sigma-Aldrich, St. Louis, MO, USA) by employing sodium borohydride 10 mM (NaBH_4_, by Sigma-Aldrich) and adopting trisodium citrate 25 mM (TSC, by Sigma-Aldrich) as the stabilizing agent. 

The typical procedure was as follows: the required volumes (see Table S1) of freshly prepared aqueous solutions containing NaBH_4_ and TSC were mixed in an ice-bath, under dark conditions. Then, the appropriate volume (see Table S1) of AgNO_3_ was added dropwise to the previous mixture under vigorous stirring for 2 h. Conversely, for the preparation of the other diluted Ag colors, a milder reducing agent was used, such as ascorbic acid 100 mM (AA, by Sigma-Aldrich), instead of NaBH_4_. In this case, the reaction was conducted in aqueous polyvinylpyrrolidone 1 %wt (PVP, $\bar{Mw}\;\;$55 kDa, by Sigma-Aldrich, as capping/stabilizing agent), by consecutively adding the suitable amounts (see Table S1) of yellow seeds (6.3 μM), TSC, AA and the dropwise addition of silver nitrate. The reaction was conducted at 50 °C, in dark conditions, under vigorous stirring for 10 min. 

In order to concentrate the Ag systems, the same synthetic routes were adopted by using more concentrated reagents (see Table 1) and also by exploiting polyvinyl alcohol (PVA, $\bar{Mw}\;$ 61 kDa, by Sigma-Aldrich) as an alternative templating/stabilizing agent to PVP.

### 2.2. Ag NPs Physico-Chemical Characterizations

UV/Vis spectra in the range between 800 and 300 nm were recorded by using UV/Vis SHIMADZU UV 2600 spectrophotometer (Shimadzu, Kyoto, Japan).

Silver nanoparticle dimensions were studied by both dynamic light scattering (DLS) analyses and transmission electron microscopy (TEM) images. For the former technique, analyses were carried out on concentrated samples, after a 1:100 dilution (this step does not affect the correctness of the obtained results, as already reported in the literature [19,20,21]), using a Malvern Zetasizer NANO ZS (Malvern Panalytical, Malvern, UK; at 25 °C). Measurements were performed on two different aliquots, for 30 scans each. Conversely, for the latter, TEM analyses were performed on LIBRA 200 EFTEM (Zeiss, Jena, Germany) instrument operated at 200 kV accelerating voltage. The TEM grids were prepared, dropping the dispersed suspension of nanoparticles in isopropanol onto a holey-carbon supported copper grid and drying it in air at room temperature overnight.

X-ray diffraction (XRD) analyses were carried out to investigate the possible crystallinity of the as-prepared Ag nanoparticles by depositing and letting the relative sols dry on the sample holder. The measurements were performed on a Miniflex 600 (Rigaku Corporation, Tokyo, Japan) Bragg–Brentano diffractometer. We used graphite-monochromated Cu K_α_ radiation (Cu K_α1_ *λ* = 1.54056 Å, K_α2_ *λ* = 1.54433 Å); diffraction patterns were collected between 30° and 80° with a step size of 0.02°.

Once stable Ag concentrated sols had been obtained, they were deposited on a crown-treated PET film by diluting them with 5 %wt of an acrylic polymers emulsion (26 % dry, Picassian^®^AC-169, purchased by Stahl Polymers, St. Peabody, MA, USA) using a doctor-blade with a thickness of 30 μm. The obtained thick films were allowed to dry in oven at 60 °C for 30 min. The chromatic coordinates were calculated according to the Commission Internationale d’Eclairage (CIELab method) [22], starting from diffuse reflectance spectra of deposited Ag sols acquired in the UV/Vis spectral range from 800 to 400 nm (SHIMADZU UV 2600 spectrophotometer, Japan). The global color differences (∆E*) were calculated according to Equation (1):

(1) $$\Delta E*=\sqrt{{\Delta L*}^{2}+{\Delta a*}^{2}+{\Delta b*}^{2}}$$

where L*, a* and b* are the lightness (0 for black, −100 for white), the red–green (positive for red and negative for green) and the yellow–blue (positive for yellow and negative for blue) components, respectively. According to the literature, no significant variation occurs when ΔE* < 5 [22,23]. In order to evaluate the possible color change, UV accelerated aging tests were performed by exposing the films under UV irradiations (Jesosil HG 500 W, λ 300–450 nm, with an effective power density of 57 mW cm^−2^) for 90 h. 

FT-IR analyses were performed on the films before and after UV aging using a Spectrum 100 spectrophotometer (Perkin-Elmer, Waltham, MA, USA) in attenuated total reflection (ATR) mode using a resolution of 4.0 and 256 scans, in a range of wavenumber between 4000 and 400 cm^−1^. A single-bounce diamond crystal was used with an incidence angle of 45°. Spectra were normalized with respect to the carbonyl peak, being the most intense one.

## 3. Results and Discussion

### 3.1. Diluted and Concentrated Ag Sols

Starting from the well-known synthetic routes [1], Ag diluted sols were easily obtained through the reduction of silver nitrate by either sodium borohydride to form yellow NPs, as clearly visible from the maximum of UV/Vis spectrum at 390 nm (Supplementary Materials Figure S1), or ascorbic acid (a milder reducing agent, Table S1) for red, blue and green sols. For the latter ones, indeed, absorption spectroscopy revealed the presence of two peaks: one centered at around 410 nm, ascribable to the presence of yellow NPs, and the other differently positioned at 510, 550 and 620 nm, respectively, for red, blue and green NPs. As reported in Table S1, the addition of trisodium citrate was necessary to obtain all the above-described colored sols; in particularly, only in the case of the yellow spherical Ag NPs, its presence was enough to prepare a stable system. Conversely for the other three cases, the addition of 1 %wt PVP was mandatory to achieve the desired size and conformation of the final nanoparticles, together with the addition of the yellow seeds for the further growth of polyhedral structures.

**Figure 1:**
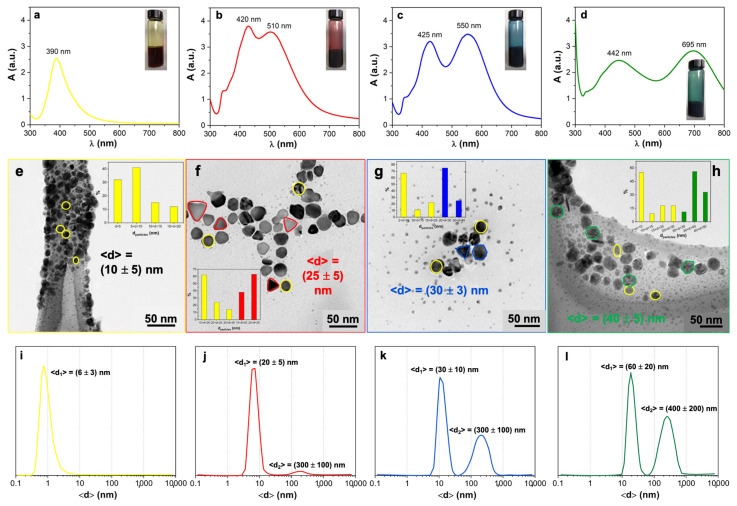
(**a**–**d**) UV/Vis spectra relative to PVP-based concentrated Ag sols (insets: photos of the corresponding synthesized colors). (**e**–**h**) TEM images together with computed particles size distribution, and (**i**–**l**) dynamic light scattering (DLS) curves by volume, in which average silver nanoparticle (Ag NP) size was evidenced.

Specifically, as stated by Khodashenas et al. [1], the silver ions promote the dissolution of Ag^0^ seeds and the subsequent transformation into triangular or polyhedral nanoprisms, according to Ostwald ripening mechanism [10]. Therefore, the correct balance among the salt precursor, the seeds and the stabilizing/capping agent content plays a critical role for the synthesis of the differently shaped anisotropic silver products. To further investigate the diluted systems, dynamic light scattering (DLS) analyses were performed, evidencing the presence of a well-defined single peak centered at around 1, 5, 7 and 10 nm for yellow, red, blue and green Ag sols, respectively (Supplementary Materials Figure S2). Hence, once the diluted silver NPs with a concentration in the range of ca. 1 and 6 μM (10th column in Table S1) had been obtained, attention was focused on further concentrating them by up to one hundred times (see 11th column in Table 1). Indeed, the novelty of the present study concerns the possibility of laying the Ag NPs onto polymeric films, resulting in micrometric thick colored layers to be used in a wide range of application fields. However, to achieve this target, sufficient concentrated sols must be synthesized so that the colors remain clearly visible, even when deposited as a film. Therefore, tailor-made synthetic routes were adopted by simply using more concentrated reagents, exploiting PVP or PVA polymers as stabilizing agents and adjusting the relative ratios among the adopted reagents (Table 1). The effectiveness of this method was confirmed by UV/Vis spectroscopy. Notably, with either PVP (Figure 1a–d) or PVA (Supplementary Materials Figure S3) structuring agents, we successfully obtained absorption spectra very similar to those recorded for diluted samples, showing almost the same maxima wavelengths. However, for red, blue and green concentrated sols, it is noteworthy to consider the higher relative intensity of the second peak (λ > 500 nm) with respect to the first one. This may corroborate the effective larger concentration of silver NPs [11]. Additionally, X-ray diffraction analyses were carried out to establish the crystallinity degree of the concentrated nano-silver. As depicted in Supplementary Materials Figure S4, all the obtained NPs showed the main silver diffraction peaks (especially the 1 1 1–100% intensity at ca. 38°) [24].

Furthermore, the stability of the as-synthesized systems was investigated by keeping the closed vials under controlled temperature of (25 ± 2) °C for seven days. Subsequently, UV/Vis spectra were recorded again, revealing that blue and green PVA-based sols were not stable, since the corresponding spectra drastically changed (see Figure S3b,c). On the contrary, complete stability of yellow and red sols was observed, as well as the PVP-based Ag colors. Specifically, for the latter, a stability period of over seven months was demonstrated, as observable in Supplementary Materials Figure S5. Indeed, by considering the absorption values, relative to the peaks in the range 390–440 nm, a negligible decrease in the intensities was noticed, as reported for yellow NPs in Supplementary Materials Figure S5b (as a representative case). As such, we assessed that PVP-based concentrated silver sols better suited our final goal. Thus, to deeply study our systems, both TEM and DLS analyses were carried out. Figure 1e–h show, as expected, the presence of spherical Ag nanoparticles smaller than 10 nm for yellow sol, whereas red, blue and green systems exhibit the presence of nano-polyhedral structures, having dimensions progressively greater (from 25 nm of red to 40 nm of green sols). Remarkably, the same trend was corroborated by the DLS analyses reported in Figure 1i–l. Interestingly, red, blue and green silver DLS curves possess two peaks: one centered at more or less the same size perceivable in TEM images; the other as a broad band at higher values (around 300–400 nm). This second peak progressively increases in relative percentage from the red sol to the green one, probably due to the formation of grape-shaped NP flocs.

### 3.2. Coloured Ag Micrometric Films

**Figure 2:**
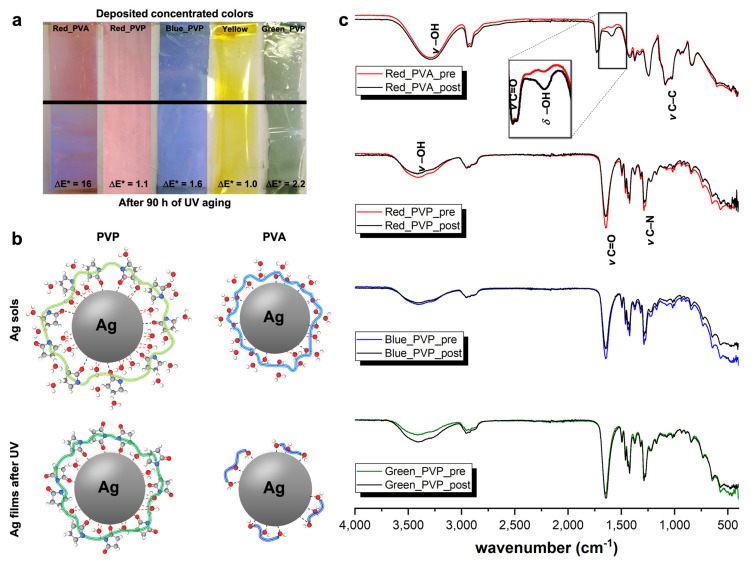
(**a**) Deposited Ag sols on crown-treated PET films showing the eventual color variation (ΔE*) upon 90 h UV treatment (lower part). (**b**) Diagram representing both PVA- and PVP-Ag interactions, also upon long-term UV exposure. (**c**) Comparison of FT-IR spectra relative to Red_PVA, Red_PVP, Blue_PVP and Green_PVP colored films, before and after UV aging test. Inset: magnified area between ca. 1700 and 1400 cm^−1^ evidencing the difference in FT-IR spectra features only for Red_PVA sample.

Once the optimized concentrated Ag colored sols had been obtained, they were diluted with an acrylic polymer emulsion at 5 %wt and then deposited onto crown-treated PET films by doctor-blading (30 μm of thickness; see Figure 2a). To assess the effective color of the as-prepared films, diffuse reflectance spectroscopy was carried out. In particular, the chromatic coordinates (L*, a* and b*) were calculated according to the already reported CIELab method [25,26]. The relative diffuse reflectance spectra are reported in Supplementary Materials Figure S6, while the computed values are displayed in Supplementary Materials Table S2 which are in line with the expected ones for yellow, red, blue and green colors. As such, we have successfully satisfied the goal of easily creating films with different desired colors.

Nevertheless, we also investigated their stability under stressed conditions. Hence, UV light prolonged exposure (90 h) was performed. The same test was carried out on the deposited PVA-red sol since, as previously discussed, it was one of the most stable systems. After UV-treatment, CIELab parameters were recalculated to compute ΔE* values. Indeed, it is reported that no significant color variation occurs when the ΔE* is lower than 5 [22]. Interestingly, all the PVP-based silver systems were stable, even after an accelerated aging test; on the contrary, the Red_PVA sample clearly exhibited a color change, resulting in a bluish shade (showing a ΔE* value of 16; see Figure 2a).

This is probably ascribable to the different chemical structures, and thus, specific adsorption, of the two adopted polymeric materials both in liquid and solid states. Indeed, Kyrychenko et al. [14,15] unveiled—via molecular dynamics simulations—the role played by either PVP or PVA in the water protective efficiency in an aqueous medium, hindering NP aggregation. In particular, concerning the former, they found that PVP wraps mainly around the metal nanoparticles through carbonyl oxygen atoms, rather than pyrrolidone nitrogen, with its backbone conformation approaching a flat-on structure [15]. Moreover, according to their theoretical studies [14,15], the PVP coating thickness may be around 0.5 nm, being slightly greater than the one formed by polyvinyl alcohol (of ca. 0.3 nm, as visible in Figure 2b, before UV treatment). Indeed, they demonstrated that using a PVP polymer with a quite low average molecular weight, i.e., $\bar{Mw}$ of 55,000 Da can lead to maintaining a highly swelled stabilizing layer around the noble metal NPs. On the contrary, given the presence of –OH moieties, PVA macromolecules cover the nanoparticles interacting via multiple non-covalent bonds through hydroxyl terminations [14]. Furthermore, according to Kyrychenko et al. [14], the adoption of 61,000 Da $\bar{Mw}$ is sufficient to shield almost 90% of the water molecules, resulting in almost the complete prevention of their penetration through the PVA coating (Figure 2b). Hence, the fact that the polymer crown is different, i.e., slightly thicker in the case of the PVP compound, can explain the lower stability of PVA-based colored sols.

Turning now to the solid state case after UV exposure, as observed by Louie et al. [27], PVP degradation at the beginning mainly proceeds through oxidation of the pyrrolidone ring, leading to the formation of imides. In this case, the PVP chains may not be broken, still guaranteeing sufficient steric NP shielding (Figure 2b, after UV treatment). Therefore, no appreciable color variation can be noticed, as in our case. On the other side, PVA degradation starts with the formation of radicals on the -CH- groups, which eventually leads to polymer degradation, thus, lowering its molecular weight (Figure 2b, after UV treatment) and forming aldehydes and ketones as terminal groups of the polymeric chains [28]. Hence, once the polymer is degraded, the macromolecules are not big enough to efficiently cover the nanoparticles surface, and tend to aggregate with each other, destabilizing the system itself. This may have caused the color variation highlighted by the high ΔE* value of 16 (Figure 2a). In order to corroborate these observations, FT-IR analyses were performed. Concerning the PVP-treated films (Figure 2c), several peaks can be ascribable to: (i) -OH stretching (broad band at 3400 cm^−1^); (ii) asymmetric *ν* -CH_2_ of pyrrole ring and symmetric *ν* -CH_2_ of the chain, respectively, at 2950 and 2920 cm^−1^; (iii) stretching of C=O at 1650 cm^−1^; (iv) bending of -CH/wagging of -CH_2_ at 1400 cm^−1^ and *ν*(C-N) at 1280 cm^−1^ [29,30]. Specifically, in the case of the blue samples, the spectra before and after UV irradiation are almost identical, indicating that no significant chemical degradation occurred. The same holds true for the red samples, with just minor variations on some peaks, related to C-H bonds. In the case of the green color, after irradiation, an increase in the intensity of the -OH stretching band is visible, probably due to a higher moisture retained by PVP, whereas the other peaks maintain the same intensity. As regards the Red_PVA sample, the peaks related to the -OH and -CH stretching modes lie at around the same position as those relative to the PVP polymer (at 3300 and 2950 cm^−1^, respectively). The double peak of C=O groups (at about 1750–1735 cm^−1^) is typical of PVAs with a high degree of hydrolysis and confirms that only a few acetate groups are present along the polymeric chains [29,30,31]. Moreover, the peak at around 1150 cm^-1^ could be due both to symmetric C-C stretching or to the stretching of hydrogen bonds arising from intramolecular interactions that occur when two -OH groups present on the same side of the plane are close enough [32]. Very interestingly, after UV irradiation, the biggest difference between the two spectra was due to the increase in the water -OH scissoring peak at about 1600 cm^−1^ (magnification in the inset of Figure 2c), clearly indicating the presence of a much higher moisture content in the sample. This could be due to the degradation of PVA, leading to the formation a high number of -OH terminal groups further favoring moisture uptake.

## 4. Conclusions

Herein, we proposed an innovative and easy method to develop either highly concentrated (up to 330 μM) colored Ag aqueous sols or thick colored films, to be applied in several research fields, by simply tailoring the capping polymeric material, namely PVP or PVA. In both cases, great stability was achieved by adopting polyvinyl pyrrolidone, thanks to its ability to form a durable nanometric crown around Ag NPs, even upon UV exposure (ΔE* below the threshold value of five). On the contrary, the possible UV-assisted chemical degradation of PVA macromolecules limited the stability of the dried film, giving rise to bigger NPs aggregates and, subsequently, color changes. This observation was fully corroborated by vibrational spectroscopy, which highlighted the presence of a greater content of hydroxyl terminal groups, confirming the partial degradation of the PVA crown, after UV treatment.

## Figures and Tables

**Table 1:** Final concentrations of reagents used for the synthesis of concentrated colors. For yellow sol: T = 2 °C and reaction time = 2 h, under dark conditions. For red, blue and green colors, the adopted yellow seeds concentration is 63 μM; T = 50 °C, reaction time = 10 min, under dark conditions. PVP = polyvinylpyrrolidone; PVA = polyvinyl alcohol; TSC = trisodium citrate; AA = ascorbic acid.

Sample		H_2_O (mL)	PVP $\bar{Mw}\;55\;\mathrm{kDa}$ (%wt)	PVA $\bar{Mw}\;61\;\mathrm{kDa}\;(\%\mathrm{wt})$	TSC (mM)	AgNO_3_ (mM)	NaBH_4_ (mM)	Seeds (μM)	AA (mM)	Ag^0^ (μM)
**One-step**	Yellow	18.7	−	−	2.5	2.5	3.0	−	−	63
**Two-step**	Red_PVP	15.0	20.0	−	6.1	4.1	−	6.83	4.3	190
	Red_PVA	15.0	−	10.0	6.9	4.1	−	6.83	4.3	190
	Blue_PVP	15.0	10.0	−	6.4	5.7	−	3.60	4.3	330
	Blue_PVA	15.0	−	10.0	6.5	5.7	−	3.60	4.3	330
	Green_PVP	15.0	20.0	−	6.7	4.9	−	1.84	4.5	225
	Green_PVA	15.0	−	10.0	6.7	4.9	−	1.84	4.5	225

## Data Availability

Data are contained within the article or supplementary material.

## Supplementary Materials

The following are available online at https://www.mdpi.com/article/10.3390/nano11040980/s1, Table S1: Final concentrations of reagents used for the synthesis of diluted colors; Table S2: CIELab color assessment for concentrated PVP-based colors; Figure S1: UV/Vis spectra relative to diluted Ag sols together with the corresponding photos; Figure S2: Dynamic light scattering data together with NPs dimensions for diluted (a) yellow, (b) red, (c) blue and (d) green colors; Figure S3: UV/Vis spectra of concentrated colors, obtained using PVA as stabilizing agent both immediately after the synthesis (continuous line) and after seven days (dotted line), keeping the closed vials under controlled temperature of (25 ± 2) °C; Figure S4: XRD spectra of the four dried concentrated Ag colors; Figure S5: (a) PVP-based Ag concentrated sols stability over time determined through UV/Vis spectroscopy and (b) UV/Vis spectra variation over time (for a total of 7 months) for the concentrated yellow sol (as a representative sample); Figure S6: DRS data of deposited concentrated colored films recorded to evaluate CIELab coordinates.
