# Supplementary material for: Stable Coloured Micrometric Films from Highly Concentrated Nano-Silver Sols: The Role of the Stabilizing Agents
Source: Nanomaterials (Basel). 2021 Apr 10;11(4):980. doi: 10.3390/nano11040980 (PMC8069504; doi:10.3390/nano11040980)
Supplement: Supplementary file 1 [file nanomaterials-11-00980-s001.pdf]

nanomaterials

*Supplementary Materials*

# Stable coloured micrometric films from highly concentrated nano-silver sols: the role of the stabilizing agents

**E. Pargoletti [1,2], M.A. Ortenzi [1,2,3] and G. Cappelletti [1,2,3,*]**

[1] Dipartimento di Chimica, Università degli Studi di Milano, Via Golgi 19, 20133 Milan, Italy; eleonora.pargoletti@unimi.it (E.P.); marco.ortenzi@unimi.it (M.A.O.)
[2] Consorzio Interuniversitario per la Scienza e Tecnologia dei Materiali (INSTM), Via Giusti 9, 50121, Firenze, Italy
[3] CRC Materiali Polimerici "LaMPo", Dipartimento di Chimica, Università degli Studi di Milano, Via Golgi 19, 20133 Milano, Italy
* Correspondence: giuseppe.cappelletti@unimi.it (G.C.); Tel.: +39 0250314228 (G.C.)

**Table S1.** Final concentrations of reagents used for the synthesis of diluted colours. For yellow sol: T = 2 °C and reaction time = 2 h, under dark conditions. For red, blue and green colours, the adopted yellow seeds concentration is 6.3 µM; T = 50 °C, reaction time = 10 min, under dark conditions.

| Sample | | $H_2O$ (mL) | PVP $\overline{Mw}$ 55 kDa (%wt) | [TSC] (mM) | [$AgNO_3$] (mM) | [$NaBH_4$] (mM) | [Seeds] (µM) | [AA] (mM) | [$Ag^0$] (µM) |
|---|---|---|---|---|---|---|---|---|---|
| **One-step** | Yellow | 18.7 | – | 0.25 | 0.25 | 0.30 | – | – | 6.3 |
| **Two-Step** | Red | 30.0 | 1.0 | 0.70 | 0.10 | – | 0.23 | 0.47 | 1.2 |
| | Blue | 30.0 | 1.0 | 0.70 | 0.23 | – | 0.12 | 0.47 | 5.4 |
| | Green | 30.0 | 1.0 | 0.70 | 0.23 | – | 0.06 | 0.47 | 5.5 |

**Table S2.** CIELab colour assessment for concentrated PVP-based colours.

| Sample | Coloured films on PET | | |
|---|---|---|---|
| | L* | a* | b* |
| Yellow | 90.1 | 4.0 | 46.6 |
| Red_PVP | 38.3 | 65.0 | 27.7 |
| Blue_PVP | 36.3 | -9.0 | -40.0 |
| Green_PVP | 56.6 | -30.0 | 21.0 |

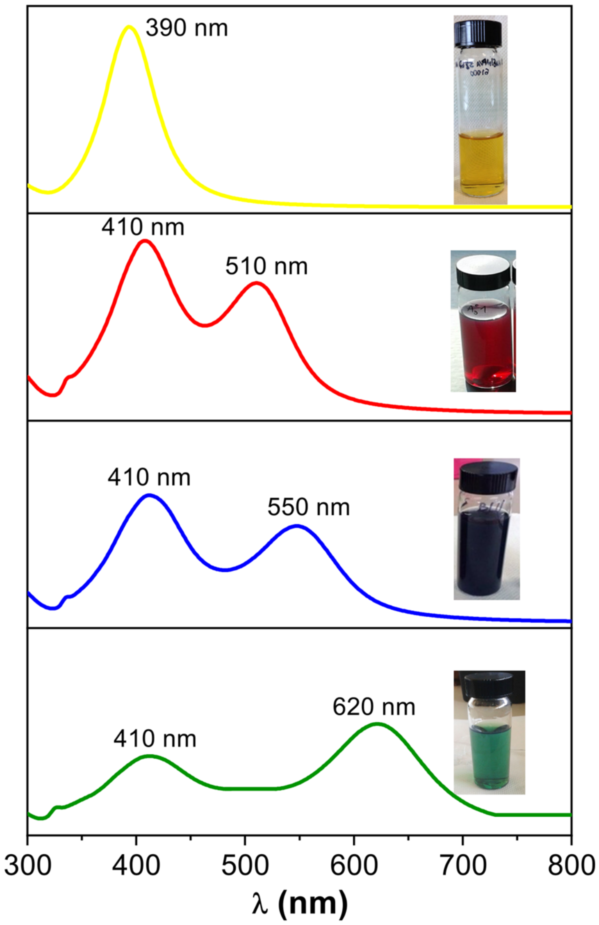


**Figure S1.** UV/Vis spectra relative to diluted Ag sols together with the corresponding photos.

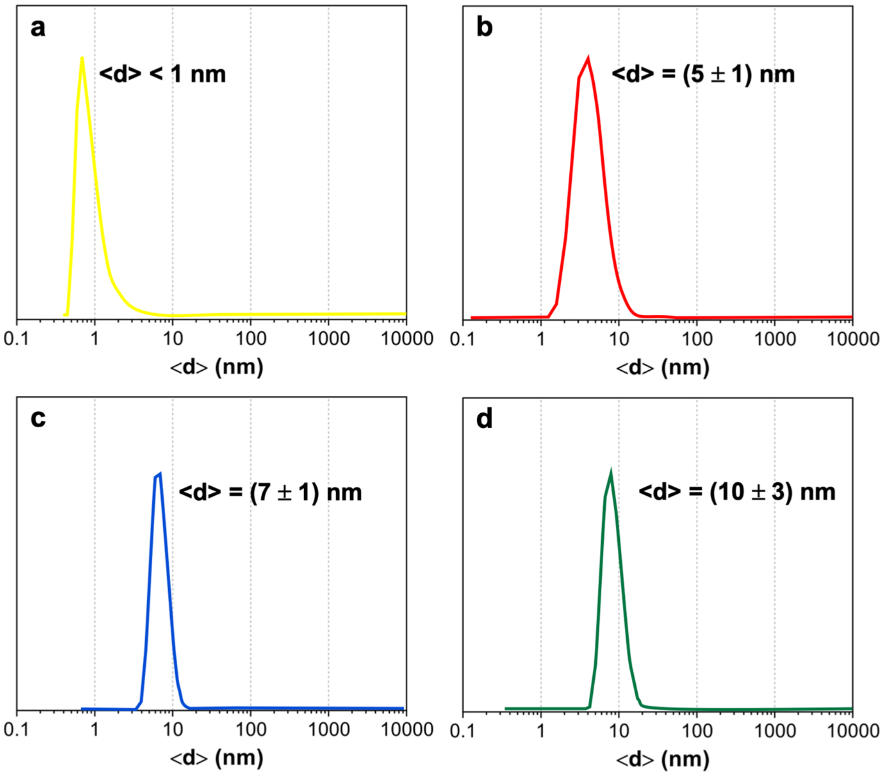


**Figure S2.** Dynamic light scattering data by volume together with NPs dimensions for diluted (a) yellow, (b) red, (c) blue and (d) green colours.

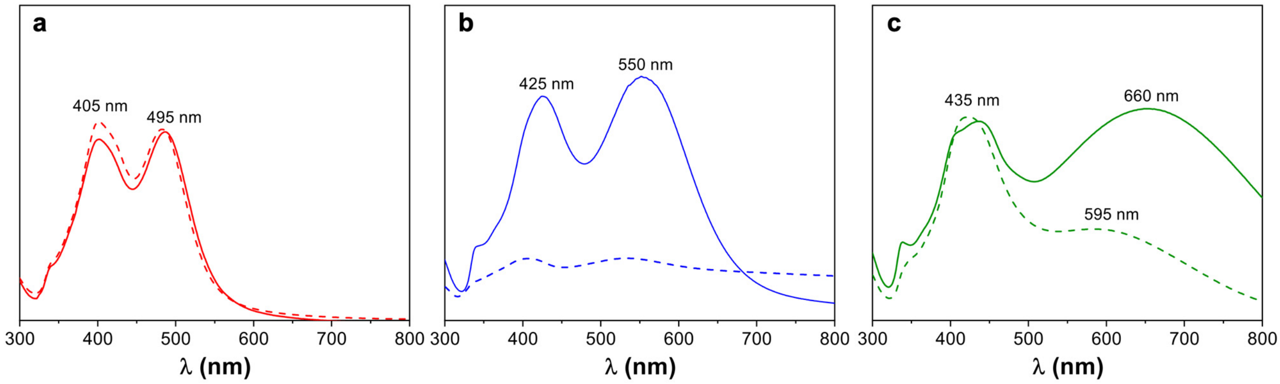


**Figure S3.** UV/Vis spectra of concentrated colours obtained using PVA as stabilizing agent both immediately after the synthesis (continuous line) and after seven days (dotted line), keeping the closed vials under controlled temperature of (25 ± 2) °C.

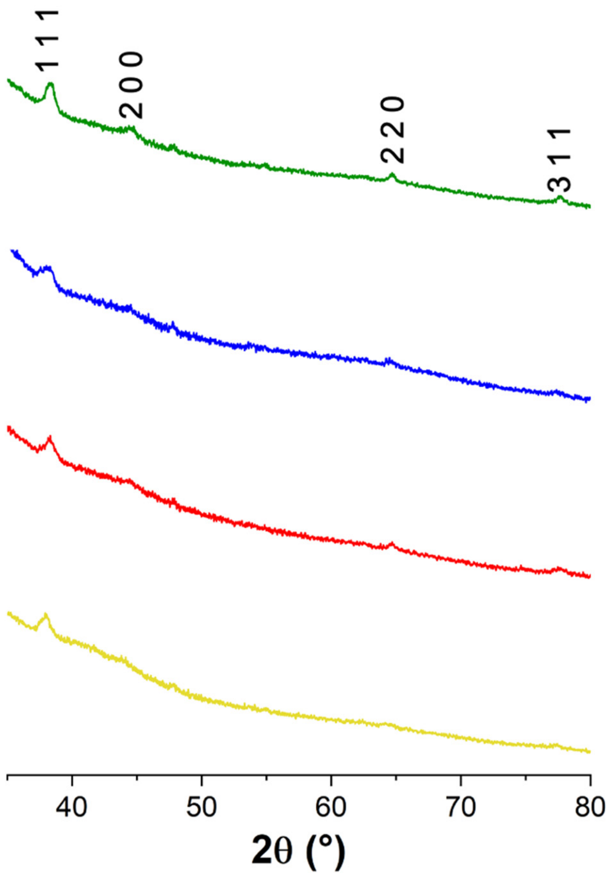


**Figure S4.** XRD spectra relative to the four dried concentrated Ag colours. The Miller's indexes (h k l) corresponding to the main silver diffraction peaks (JCPDS N. 04-0783) have been highlighted.

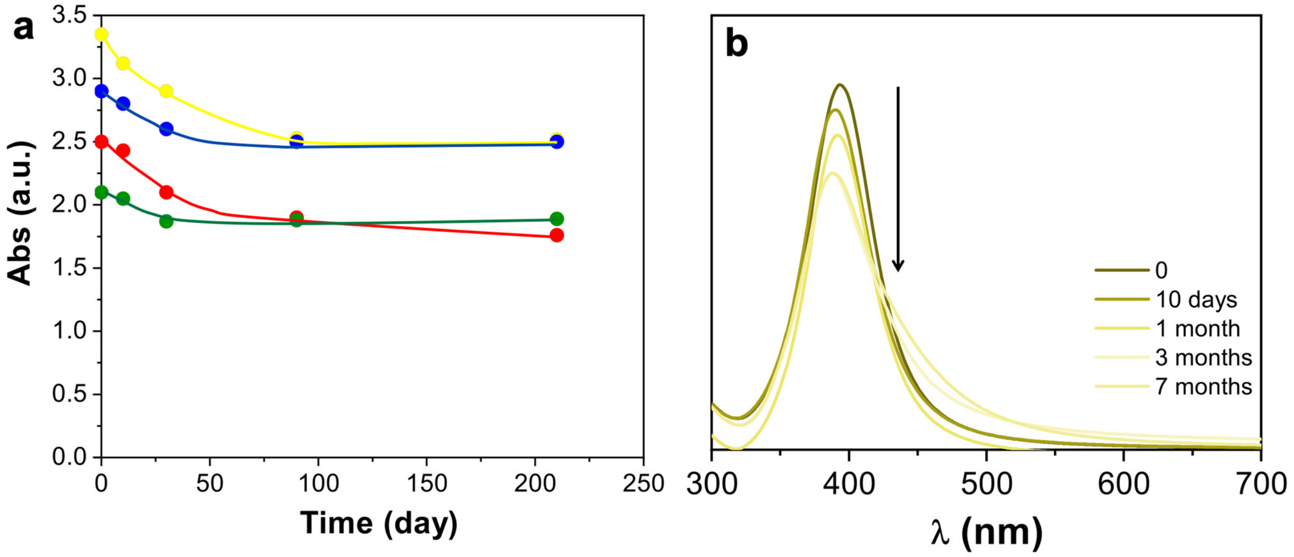


**Figure S5.** (a) PVP-based Ag concentrated sols stability over time determined through UV/Vis spectroscopy (for each colour the considered absorbance values are relative to the first band in the range of 390-440 nm). (b) UV/Vis spectra variation over time (for a total of 7 months) for the concentrated yellow sol (as a representative sample).

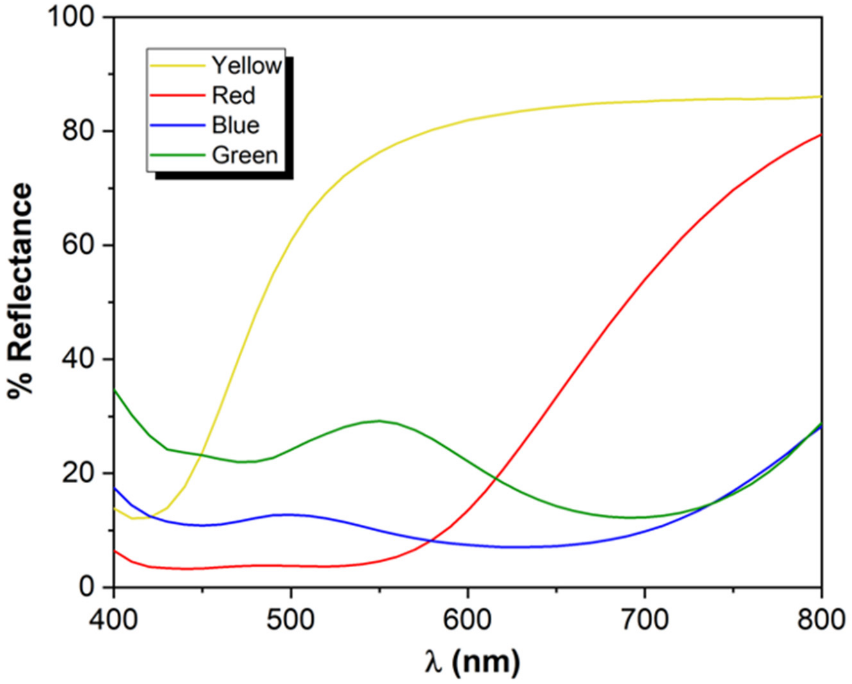


**Figure S6.** Diffuse reflectance spectra (DRS) of deposited concentrated coloured films recorded to evaluate CIELab coordinates.
